# Progress in Medicine

**Published:** 1899-07-15

**Authors:** 


### Progress in Medicine.

### NEUROLOGY.

**Aphasia.**—Collier[1] records a case in which complete destruction of Broca's convolution by a neoplasm in a right-handed person produced no aphasia. The patient, a woman of 23 years of age, was admitted very late in the disease in a semicomatose condition. There was no word-blindnesss or word-deafness. She answered simple questions, and said spontaneously, "Why can't you leave me alone?" "Let me go to sleep; I'm all right." She could write her name. Paralysis of both sixth nerves was the only palsy present. Optic neuritis was present in both eyes. At the post-mortem examination a growth was found involving the inferior margin of the left second frontal convolution at about its middle, the whole posterior part of the left third frontal convolution, the posterior part of the orbital lobule. and the anterior gyrus of the island of Reil. The right hemisphere was quite healthy. This case proves the possibility of the fortuitous major activity of the motor speech centre in the right third frontal convolution in a right-handed person. It also shows that right-handedness does not necessarily involve the major activity of the third left frontal convolution for the production of articulate speech. It has been suggested by Bramwell that in some right-handed persons whose ancestors were left-handed the motor speech centre may possibly be situated in the right hemisphere. Such an explanation does not hold good in this case, as there was no instance of left-handedness in the family. The facts of the case suggest that the unusual location of the leading or active motor speech centre in the right hemisphere and the presence of a neoplasm in the left third frontal convolution were simply coincident, and that the right-third frontal convolution had always performed the function of a motor speech centre. The converse of the case is known, for Dickinson and Wadham have recorded instances showing that left-handedness does not always transfer the leading speech centres to the right side of the brain.

Bramwell[2] has recorded a case of aphasia and right-sided hemiplegia in a left-handed-man. The patient, who came of right-handed ancestors, was left-handed for everything except writing, which he had been taught with some difficulty to do with his right hand. The illness, which was of sudden onset, and probably vascular in nature, caused right hemiplegia and aphasia (partly motor and partly sensory) of an enduring character. The conclusion arrived at was that, in this left-handed man, the speech centres were situated in the left side of the brain. Bramwell thinks that this may have been due partly to heredity—the man's ancestors were right-handed—and partly to the patient's having been taught to write with the right hand. He is of opinion that Collier's case, recorded above, of a tumour of the left motor speech centre in a right-handed person, may admit of another explanation than that advanced by Collier (which was that in that individual the motor speech centre was on the right side). Bramwell thinks that it may be accounted for by supposing that as Broca's convolution was gradually destroyed by the neoplasm substitution by the right side took place. Such a view would harmonise with the well-known fact that an ordinary cortical motor centre—that for the hand, for example—can be completely destroyed by a neoplasm without the production of any paralysis. Collier's view, that the leading motor speech centre may exceptionally be situated in the right hemisphere in right-handed persons, can only be considered proved by observation of a sudden—vascular—lesion of Broca's convolution not producing asphasia in a right-handed individual.

**Tumours.**—Tumours of the cerebellum, optic thalamus, and frontal lobe have certain features in common which cause them to resemble each other to a variable degree in different cases. Their differential diagnosis has recently been discussed by Risien Russell.[3] The existence of headache, vomiting, and optic neuritis may be left out of consideration as common to all forms of intracranial tumour. The special features which are common to tumours in the above-mentioned three situations are ataxy, the characteristic attitude, nystagmus, anosmia, absence of knee jerks, mental defects, and loss of control over the sphincters.

Inco-ordination is not met with nearly so commonly in cases of tumour of the frontal region and optic thalamus as in those of the cerebellum, and if present never reaches such a degree of intensity as it may do in cerebellar tumours. Moreover, the type of inco-ordination is not the same. In cerebellar tumours there is a variable degree of general titubation, in which the head, neck, and trunk share. In frontal cases, while the unsteadiness of gait and falling in some one or other direction may be features, neither general titubation nor inco-ordination of individual limbs is so manifest. In tumours of the optic thalamus the inco-ordination generally manifests itself in some individual limb in the shape of choreiform or athetoid movements rather than by ataxy of gait.

The attitude in tumours of the cerebellum is characteristic; there is an approximation of the side of the face to the shoulder on the side of the lesion, whilst the head is at the same time rotated on its vertical axis so that the chin points to the normal side. On the other hand, no characteristic attitude has been observed in tumours of the frontal region or of the optic thalamus. Nystagmus is much more common and pronounced in tumours of the cerebellum than in growths of the other two situations, and whereas it may be either spontaneous or evoked only on voluntary displacement of the globes in cerebellar cases, it is usually the latter variety only which is found in tumours of the frontal region and optic thalamus. Anosmia may be found in cases of both cerebellar and frontal tumours, but is not found in those of the thalamus. Absence of the knee jerks was at one time thought to be characteristic of cerebellar and frontal tumours, but it is now recognised that any large neoplasm within the cranial cavity, especially if it be one of rapid growth, is attended with great diminution or abolition of the knee jerks. Mental defect may be met with in the later stages of a tumour of the brain irrespective of its precise situation, but if it occurs early in the clinical course of a given case, it is strongly in favour of the tumour being situated in the frontal lobe. The mental defect is not of any one type, though a peculiar hilarious state is common, and loss of memory is usually prominent, and a dull apathetic state is apt to come on. Some cases of tumour of the optic thalamus may present somewhat similar mental defects, but these form no part of the clinical picture of a case of cerebellar tumour. Aphasic defects are very rare even in cases of frontal tumour, which invade Broca's convolution, probably owing to the slowness of invasion permitting of substitution; but if they occur, the tumour is in the left frontal region. Loss of control over the sphincters may occur in frontal and thalamic tumours, but not in those of the cerebellum; probably it is not due to any paralytic defect of the muscles, but rather the result of the mental hebetude which is so common in the first two classes of tumours. Fits are in favour of the tumour being in the cerebrum. Cerebellar disease may exceptionally give rise to convulsive attacks, but these resemble tetanus seizures, as a rule, rather than convulsions of cerebral origin. Both general and Jacksonian fits may be met with in tumours of both thalamus and frontal lobe. Hemiplegia may be met with in tumours of the optic thalamus and of the frontal lobe owing to extension of the growth, and in both cases the arm is more prone to suffer than the leg. Hemiparesis is rare in cerebellar affections, except when these are acute, such as abscess.

Tumours of the frontal lobe and of the optic thalamus may give rise to facial paralysis of cerebral type, *i.e.*, affecting only the lower half of the face, whereas if cerebellar tumours cause facial paralysis it is always of the peripheral type. Tumours of the optic thalamus and frontal region may also, like tumours in any part of the brain, give rise to paralysis or more commonly paresis of the facial nerve of a peripheral type, a result apparently of the general increase of intracranial pressure on the nerves. Deafness of a variable degree may result from tumours of the frontal lobe or optic thalamus as a result of general increase of intracranial, but it occurs with greatest frequency and severity in tumours of the cerebellum, this defect, like facial paralysis, only occurring when the tumour is in the anterior part of the posterior fossa.

Intense double optic neuritis coming on quickly is characteristic of cerebellar tumours; tumours of the frontal region and thalamus also give rise to double optic neuritis; but inequality is very often marked in frontal tumours, the neuritis being greater in degree on the same side as the neoplasm. Hemianæsthesia and hemiopia may occasionally result from tumours of the thalamus spreading into the posterior part of the internal capsule. Paresis or paralysis of ocular muscles may be brought about by pressure on the base of the brain in cerebellar tumours. Tumours of the frontal may cause paralysis of eye movements by spreading backwards to the centres in the middle frontal convolution, but very rarely, as the lesion is readily compensated for.

**Locomotor Ataxy.**—Kende[4] comes to the following conclusions in regard to the etiology of locomotor ataxy. Syphilis is not the only true cause, and it can be shown in many cases that it does not even act as a predisposing cause. The opinion that cures would result if the inunction methods of treatment were carried out more systematically is not true; on the contrary, inunctions are apt to do more harm than good in cases of simple tabes; and in those cases in which cures have been reported by these means of treatment it is probable that the diagnosis was doubtful. Tabes is more common among civilised than among primitive peoples. It has probably at its foundation some developmental defect of the nervous system, or it may be an acquired defect due to extreme fatigue or over exertion in the struggle for existence.

Thomas[5] in a statistical study of 111 cases of tabes at the Johns Hopkins Hospital, Baltimore, found that in a large proportion of cases there was a history of syphilis, whilst this was absent in a considerable number. In negroes tabes is relatively uncommon, although this race represents a large number of syphilitics—thus confirming Kende's views quoted above. The partial immunity of women is greater than can be satisfactorily accounted for by the relative infrequency of syphilis among them. Apparently syphilis is not the only factor in the etiology of tabes. The time between syphilitic infection and the appearance of the initial symptoms of tabes varied from 2 to 30 years. Gastric crises occurred in 9 cases, optic atrophy in 11, eye paralyses in 30, Argyle Robertson pupil in 70. In 2 cases Romberg's symptom was marked in spite of the blindness. Charcot's joint disease was observed in 5 undoubted cases, and in 3 others there were suspicious enlargements.

Trümmer[6] has recorded the cases of three patients in whom there was absolutely no history or evidence of syphilis, and in whom locomotor ataxy followed a severe traumatism. The accidents were injury to one foot, injury to one arm, and a fall on the back, respectively.

Krauss[7] has recorded the case of a woman afflicted with well marked tabes, who suffered from characteristic attacks of hepatic crises. These attacks recurred every four or five weeks. The pains were deepseated, very intense, and continuous. Each attack was accompanied by jaundice, the skin and urine becoming deep yellow and the stools clay-coloured, lasting from four to five days. The hepatic area during a crisis was acutely tender. To procure relief and sleep morphia had to be injected. He gradually lost strength and wasted, the hepatic attacks becoming more and more intense. A post-mortem examination showed sclerosis of the posterior columns of the spinal cord, and a typical mottled and nutmeg liver. The bile passages were normal. No lesion in the thorax or abdomen was found to which the condition of the liver could be attributed. To the various tabetic crises of Charcot—viz., gastric, laryngeal, cardiac, and rectal—must now be added the hepatic, which, with the rare type of vesical, nephralgic, and urethral, complete the list of these painful and paroxysmal syndromes which may occur in tabes.

[1] Collier, Lancet, Mar. 25, 1899. [2] Bramwell, Lancet, June 3, 1899. [3] Risien Russell, Clin. Jour., April 12, 1899. [4] Kende, Zeitsch für Klin. Med., Vol. 28, 1899. [5] Thomas, Phil. Med. Jour., Dec. 17, 1898. [6] Trümmer, Neurol. Centralblatt, June, 1898. [7] Krauss, Jour., of Nerv. and Ment. Dis., Feb., 1899.

